# The 2009 H1N1 pandemic, vaccine-associated narcolepsy, and the politics of risk and harm

**DOI:** 10.1177/1363459320925880

**Published:** 2020-06-02

**Authors:** Venla Oikkonen

**Affiliations:** Tampere University, Finland

**Keywords:** H1N1, narcolepsy, pandemics, politics, vaccine injury

## Abstract

The article traces the emergence of a new type of vaccine injury—vaccine-associated narcolepsy—following immunization with Pandemrix vaccine during the 2009 H1N1 pandemic in Europe. The article highlights the processual nature of vaccine injury: it shows how vaccine-associated narcolepsy emerges gradually as a recognized object through epidemiological and immunological studies as well as patient organizations’ public discourses. The article argues that despite public recognition of injury, vaccine-associated narcolepsy remains an incongruous object characterized by underlying tensions. These tensions take shape in relation to the history of vaccine injury debates, on the one hand, and the connection between vaccine-associated narcolepsy and non-vaccine-related narcolepsy, on the other. The article shows how these underlying tensions enable a range of mutually incompatible framings and mobilizations through which risk, harm, responsibility, and justice are claimed and negotiated.

## Introduction

Vaccine-associated narcolepsy is a novel type of vaccine injury associated with the H1N1 vaccine Pandemrix used during the 2009 pandemic in Europe. The case is rare in the history of vaccine injury claims in that vaccine-associated narcolepsy has been established statistically on the population level. The case illuminates the complexities of vaccine injury by making visible the processes through which vaccine-associated narcolepsy emerged as a suspicion, solidified as a recognizable phenomenon, and yet remained open to multiple reconfigurations. Focusing on these processes, the article traces how scientific and public debates about vaccine-associated narcolepsy move from questions of the *realness* of injury to the *nature* of vaccine-associated narcolepsy as a biomedical condition.

The story begins in spring 2009, when a new influenza outbreak was reported in Mexico and California at the end of the usual influenza season. The virus was sequenced promptly and shown to be a novel A/H1N1 (swine flu) strain. The unexpected outbreak raised concerns (MacPhail, 2014). In June 2009, the World Health Organization (WHO) declared a pandemic following the rapid spread of new cases across continents. Yet, mortality from H1N1 appeared considerably lower than in visions of pandemic threat constructed around highly pathogenic avian influenza or SARS (Abeysinghe, 2015). This low mortality became a frame against which states’ vaccination policies and vaccine safety concerns were later evaluated.

Declaring a pandemic operated as a trigger within the international structures of pandemic preparedness. The WHO recommended mass vaccinations, pharmaceutical companies started vaccine manufacture, and many states made swift decisions about buying vaccines on the basis of preexisting agreements with the pharmaceutical industry (e.g. Abeysinghe, 2015: 102–132). Vaccine manufacture in pandemic times involved an expedited licensing process in order to make vaccines available during the pandemic peak. In Europe, eight H1N1 vaccines were licensed, and between October 2009 and August 2010 at least 38 million people in the European Union (EU) and European Economic Area (EEA) countries were immunized (European Centre for Disease Prevention and Control (ECDC), 2012). The most commonly used vaccine in Europe was GlaxoSmithKline’s (GSK) Pandemrix. In some European countries, such as Finland and Sweden, Pandemrix was the only vaccine offered in the mass vaccinations that began in October 2009 (ECDC, 2012).

While the H1N1 pandemic waned in spring 2010, a new concern arose: in Finland and Sweden, where vaccination rates were particularly high, doctors noticed an unusual number of new cases of narcolepsy among children vaccinated with Pandemrix; there were suspected cases also in Iceland (ECDC, 2011). Narcolepsy is a chronic disease whose symptoms include excessive and uncontrollable daytime sleepiness, disturbed nighttime sleep patterns, sleep hallucinations and sleep paralysis, and—in the cases associated with Pandemrix—cataplexy, a sudden loss of muscle control when experiencing strong emotions. Epidemiological studies established a connection between narcolepsy and Pandemrix among children and adolescents first in Finland and Sweden (e.g. ECDC, 2012; Nohynek et al., 2012; Persson et al., 2013), and later in several other European countries (e.g. Heier et al., 2013; Miller et al., 2013). Some further studies found an increase in narcolepsy also among adults, but the incidence was less pronounced (e.g. Stowe et al., 2016). While the contracts exempted GSK from financial responsibility, several governments have paid compensations to affected families. At the same time, vaccine-associated narcolepsy has become an object of political and societal debates, shaping pandemic preparedness plans, public attitudes toward vaccination programs, as well as patient activism around narcolepsy and medical injuries (e.g. Lundgren, 2017; Lundgren and Holmberg, 2017).

## Analytical approach

The article traces how vaccine-associated narcolepsy emerged through epidemiological surveys, immunological studies, and the public discourses of patient organizations. I call the phenomenon *vaccine-associated narcolepsy*—a term used in many biomedical articles—because the word *association* highlights the debated nature of the connection between the vaccine and narcolepsy, thereby pointing to its character as an object on the move. The article maintains that what vaccine-associated narcolepsy is not only a biomedical but also a social and political question: it sets the parameters for what kinds of legal claims can be made about vaccine injury, what kind of patient activism appears as legitimate, and how future immunization campaigns conceptualize risk and benefit.

The article begins with a discussion of the social, legal, and historical study of vaccine injuries in order to contextualize the Pandemrix debate. The following three analytical sections utilize different types of data. The first section traces the emergence of vaccine-associated narcolepsy through institutional reports and epidemiological studies by public health organizations and epidemiological research groups. The section explores the steps through which a new vaccine injury comes into being as a hazy possibility and then gradually as epidemiological reality. The second section turns to the rich biomedical literature on the causative mechanisms of vaccine-associated narcolepsy, highlighting the evasiveness of intricate material processes inside the body. This literature partly overlaps with epidemiological studies, as many scientists work in biomedical research groups while also serving as experts in epidemiological investigations for public health institutions. The third analytical section investigates the public websites of patient organizations in three affected countries: Sweden, Ireland, and the United Kingdom. This provides an illustrative contrast: while the organizations share concerns, they also tackle with unique issues arising from national public health frameworks, which set the parameters within which vaccine injuries are negotiated. I trace how the websites, including linked stories and educational materials, engage with scientific and public health accounts, mobilizing as well as mitigating tensions around the nature of vaccine-associated narcolepsy.

Methodologically, the article builds on critical textual analysis. I identify assumptions about vaccine injury across the analyzed materials, paying special attention to unresolved tensions between different accounts. I read assumptions of vaccine-associated narcolepsy against the histories of vaccine injury in order to trace continuities and abruptions and locate their effects on current imaginaries of vaccine injury. In the case of patient organization websites and linked materials, I also pay attention to how cultural narratives are mobilized to establish or reframe vaccine injury.

## Complexities of vaccine injury

Vaccine injury is a multilayered phenomenon with entangled biomedical, epidemiological, social and personal elements. Vaccines are cultural as much as technological: as Bernice Hausman aptly puts it, vaccines “circulate in networks of value and consequence—government agencies, medical researchers, manufacturers, marketing plans, medical offices, families” and “garner meaning from these networks, becoming symbols at the same time that they are material actors in them” (Hausman, 2016: 194). Vaccine injuries reflect these complexities.

As a biomedical process, vaccine injury is typically evasive. Clear cases of vaccine injury certainly exist: for example, in the 1955 Cutter Incident, improperly inactivated live polio vaccine caused over 250 cases of paralytic polio in the United States (some directly from the vaccine, others through infection from those vaccinated; Colgrove, 2006; Conis, 2015). Other times, vaccine injury may be based on a temporal proximity between immunization and the onset of symptoms, but the precise causation is not fully understood (e.g. Conis, 2015: 147; Kirkland, 2016). The association between the 1976 H1N1 influenza vaccine and Guillain-Barré syndrome (an autoimmune infection of the nervous system that results in temporary paralysis; Conis, 2015: 149; Dehner, 2012: 185–188) is one example. However, in many cases of suspected or claimed vaccine injury, the symptoms constitute a blurry object, consisting, for example, of allergies, behavioral changes, metabolic problems, or developmental delays, whose onset may or may not be connected to the vaccine (Reich, 2016). As Sharon Kaufman (2010) writes in the context of claims made by vaccine critics about the measles–mumps–rubella (MMR) vaccine and autism in the late 1990s and early 2000s, a condition like autism “represents a labeling process and a category of knowledge in which the boundaries of symptom inclusion and exclusion are fluid” (p. 11).

Theories of the causative mechanisms of vaccine injury have changed over time. This is not simply a matter of an evolving understanding of biological processes; ideas of causation are entangled with social and political contexts. During the nineteenth- and early twentieth-century small pox immunization campaigns, vaccine injuries were often pinned to the risk of introducing biological material from another species or other humans into the body (Conis, 2015: 132–134). With the rise of environmentalism in the 1960s and 1970s, risk was located in the potentially toxic vaccine ingredients (Conis, 2015: 132). In the 1990s, these concerns focused on thimerosal, a mercury-based substance used as a preservative, reflecting the growing public awareness of mercury as dangerous to humans (Conis, 2015: 150). Furthermore, the increasing number of childhood vaccines, and especially multivalent vaccines (such as MMR or DPT), has been portrayed by vaccine critics as an immune overload capable of triggering injuries (Gottlieb, 2016; Reich, 2016).

The question of vaccine injury is embedded in an unsettled tension between individual symptoms and population-level evidence. For epidemiology, vaccine injury emerges when there are enough cases to suggest a statistically significant increase. For example, in the case of the MMR vaccine, the connection between MMR and autism claimed by vaccine critics was debated fervently in epidemiological terms, focusing on whether the increase in autism diagnoses was connected to the wide use of the MMR vaccine (vaccine critics’ view), or to some other factor such as changes in diagnostic categories (a prominent medical view; Conis, 2015; Kaufman, 2010; Reich, 2016). At the same time, emphasizing population-level evidence in vaccine injury claims inevitably excludes very rare injuries, which do not reach the epidemiological threshold of recognition. Indeed, ethnographic studies of vaccine criticism have shown that vaccine-critical parents often prioritize parental intuition over population-level estimates of vaccine injury risk (Gottlieb, 2016; Reich, 2016). This involves seeing each child as immunologically and developmentally unique, with the result that the risk of vaccine injury becomes individualized (Gottlieb, 2016: 167–168; Reich, 2016: 137–138).

Vaccine injury is also a legal object. What counts as injury differs between countries and legal systems (Holland, 2018). It is also renegotiated within each legal system, as has happened, for example, in the history of the US vaccine court and important cases such as the Autism Omnibus Proceeding concluded in 2009 (Decoteau and Underman, 2015; Haertlein, 2012). As a legal object, vaccine injury is located at the intersection of the interests of private citizens, public health actors, and the pharmaceutical industry, and hence legal definitions may not fully overlap with scientific ones (Holland, 2018; Kirkland, 2016). For example, many legal systems recognize the need to compensate private citizens for injuries acquired by following public recommendations even if conclusive scientific evidence of injury is not available. Another rationale is to maintain strict standards to prevent false claims from encouraging public suspicion of vaccines. A third rationale is to ensure that compensations required from pharmaceutical companies remain moderate in order to guarantee vaccine production.

Furthermore, vaccine injuries are temporally and spatially situated. The risk of vaccine injury is perceived in relation to the likelihood and severity of the vaccine-preventable disease. The risk of vaccine injury is more likely to be accepted during a life-threatening epidemic than when the risk of illness is less acute as with diseases eliminated by vaccines (Gottlieb, 2016; Heller, 2008: 84–111; Reich, 2016). Not surprisingly, pro- and anti-vaccination campaigns portray vaccine-preventable diseases differently. For example, in the debate about hepatitis B as a required childhood vaccine in the United States in the 1980s and 1990s, vaccine proponents portrayed children as vulnerable to hepatitis B through daycare and school, while vaccine critics saw it as an illness of homosexuals and intravenous drug users (Conis, 2015: 179–202). Vaccine injuries are also conceptualized in relation to public health scandals. In the United Kingdom, the government’s initial dismissal of mad cow disease in the 1990s formed a fertile ground for public claims about the government covering up an MMR-autism connection a few years later (Stöckl and Smajdor, 2017).

The emergence of new vaccine injuries takes place in relation to these histories of suspected, proven, and unsubstantiated claims of vaccine injury. As the following sections demonstrate, these past incidents and debates set the imaginative parameters within which the connection between Pandemrix and narcolepsy was conceptualized and negotiated.

## Enacting vaccine-associated narcolepsy through epidemiology

Compared to past vaccine injury debates, the realness of Pandemrix-associated narcolepsy was established relatively quickly through epidemiological surveillance and research. In the summer of 2010, some 10 months after the mass vaccination campaigns had started, Swedish and Finnish doctors began reporting suspected cases of vaccine-related narcolepsy through international signaling systems for medicinal adverse events (the EudraVigilance system) and public health threats (the Early Warning and Response System). The reports were met with initial caution by public health actors (e.g. Briggs, 2011; Connolly, 2011; ECDC, 2011), which is not surprising considering the heated public debates in the history of vaccine injury claims. To resolve the issue, epidemiological studies were launched to determine whether vaccine-associated narcolepsy existed as a statistically significant phenomenon rather than individual events of possible adverse reaction. The long-standing tension between individual cases and population-level risk of vaccine injury, outlined in the previous section, runs through these studies. While vaccine-associated narcolepsy existed before the epidemiological studies as lived experience and doctors’ suspicions, it emerged as an epidemiologically recognizable condition only through statistical observations.

Because of the urgency of establishing or disproving the connection between Pandemrix and narcolepsy, epidemiological studies relied on retrospective approaches that utilized existing national health registries of vaccine adverse reactions, vaccination status, and narcolepsy diagnosis, as well as information derived from patient files. In Finland, a national narcolepsy task force coordinated though the National Institute for Health and Welfare published an interim report in January 2011 (Terveyden ja hyvinvoinnin laitos/National Institute for Health and Welfare (THL), 2011b) and a final report in August 2011 (THL, 2011a). In Sweden, the Medical Products Agency published a registry study comprising 57 percent of the Swedish population in March 2011 (Medical Products Agency/Läkemedelsverket (MPA), 2011a), followed by a case inventory study of vaccinated and non-vaccinated children and adolescents with narcolepsy in June 2011 (MPA, 2011b). The reports from both countries discovered a significantly heightened occurrence of narcolepsy among vaccinated children and adolescents, ranging from a 6.6-fold higher risk in Sweden to a 12.7-fold higher risk in Finland. These studies were followed by a transnational study, commissioned by the European Centre for Disease Prevention and Control (ECDC) and conducted by the Vaccine Adverse Event Surveillance and Communication Consortium VAESCO (ECDC, 2012). The study compiled and reevaluated data from the two signaling countries (countries that had reported cases) as well as six non-signaling countries (countries that had not reported cases): Denmark, Italy, France, the Netherlands, Norway, and the United Kingdom (ECDC, 2012). It confirmed the results from Finland and Sweden, but did not find evidence of vaccine-associated narcolepsy in the non-signaling countries. However, subsequent national studies have suggested that several countries—some with low vaccination rates that made statistical detection difficult—were affected, including Ireland, the United Kingdom, France, and Norway (Dauvilliers et al., 2013; Heier et al., 2013; Miller et al., 2013; O’Flanagan et al., 2014; Trogstad et al., 2017).

As in the history of vaccine injury claims, numbers played a constitutive role in the recognition of vaccine-associated narcolepsy: the accumulation of cases, registered through the statistical methods of epidemiological studies, produced a sense of movement. Reflecting the border-crossing nature of the pharmaceutical industry as well as the pandemic itself, numbers of vaccine-associated narcolepsy operated at the intersection of national and international public health. Although the epidemiological realness of vaccine-associated narcolepsy relied on national databases and the idea of national populations as biomedically meaningful entities, the existence of injury was ultimately verified through comparisons between countries. This process located vaccine-associated narcolepsy *spatially* as spanning some but not all European countries.

Transnational epidemiological research involved considerable *tinkering* (e.g. Mol et al., 2010) in order to produce a coherent, measurable phenomenon, as the national databases and health systems were asymmetrical. A central aim of the VAESCO report was to tame epistemically the not-fully-compatible analytical apparatuses in national studies. Follow-up studies, such as Miller et al.’s (2013) retrospective analysis that confirmed the incidence of Pandemrix-associated narcolepsy in England in the United Kingdom, also engaged in tinkering to adjust their research design to the different sampling methods, public health infrastructures, and case confirmation criteria in the Finnish and Swedish studies. One central question was whether all cases filled the diagnostic criteria of narcolepsy. Different doctors had used slightly different sets of diagnostic tests, the full set of tests was not always recorded in the files, or the test results were interpreted differently. Another question was how to define the onset of narcolepsy. This was important, as vaccine injury claims rely on the temporal proximity between immunization and adverse events. Relying on families’ retrospective accounts of the onset was considered unreliable, but so was relying on the date of the first medical visit, referral to a sleep specialist, or narcolepsy diagnosis, as there might be considerable and irregular delays between these events. While national studies arrived at different solutions, the VAESCO report sought to establish points of comparison. Through these comparisons, vaccine-associated narcolepsy emerged gradually as a scientifically established, transnational phenomenon.

Comparisons between national populations raised the question of why vaccine-associated narcolepsy appeared initially in only some of the countries that had relied on Pandemrix. For example, Miller et al.’s (2013) study notes that the reports from Finland and Sweden had “led to speculation that some unidentified factor was operating in these countries” (p. 3). While commentators have pointed to the likely role of differences in vaccine uptake between countries, national differences also drew attention to possible causative mechanisms. Why were the neurological and immunological systems of particular children in some countries so vulnerable? Such questions were beyond the reach of epidemiology and statistics, requiring a different set of biomedical approaches.

## Mechanisms of vaccine-associated narcolepsy

Despite its newly established realness as an epidemiological phenomenon, vaccine-associated narcolepsy remained evasive as an embodied biomedical condition. This reflected the fact that the underlying mechanisms of narcolepsy in general were blurry. Tellingly, the 2012 VAESCO report opened by stating that “[n]arcolepsy is an underdiagnosed disease of widely unknown etiology”; that is, the precise mechanisms of disease causation were unknown (ECDC, 2012: 1). In this, narcolepsy resembled conditions such as developmental delays, immune deficiencies, metabolic disorders, and autism spectrum symptoms invoked in vaccine critical discourse (e.g. Kaufman, 2010: 10–11). The situation was complicated by the fact that excessive daytime sleepiness or mood and personality changes associated with narcolepsy were easily confused with mental health issues, and thus narcolepsy might go undiagnosed or misdiagnosed. Second, narcolepsy had not been previously linked to vaccines, and thus there was little literature that researchers could build on to identify a potential biological mechanism of vaccine injury.

Following epidemiological studies, researchers turned to the material processes inside bodies. While narcolepsy is an understudied phenomenon, a set of risk factors have been established. Narcolepsy is divided into two categories: type 1 comes with cataplexy, a sudden loss of muscle control when experiencing emotions, and type 2 without cataplexy. Type 1 narcolepsy is assumed to involve an autoimmune reaction, in which the body’s immune system damages neurons involved in the functioning of hypocretin (orexin), which controls wakefulness. In type 1 narcolepsy, hypocretin levels are very low or undetectable. Patients with type 1 narcolepsy also tend to have a particular genetic marker, human leukocyte antigen (HLA) DQB1*06:02. However, the marker is very common—close to 25 percent of the world population carries it—so the allele alone is not enough to trigger narcolepsy. It is also known that narcolepsy typically appears in adolescence, suggesting that it involves the maturing neurological and immunological systems.

A central question concerned whether vaccine-associated narcolepsy was part of general, non-vaccine-related narcolepsy, or whether it constituted a distinct condition. Cases of vaccine-associated narcolepsy almost always included cataplexy and low hypocretin levels, aligning it with type 1 narcolepsy. A 2014 study by Bomfim et al. suggested that the genetic profile of non-vaccine-related and vaccine-associated narcolepsy patients was similar at key molecular loci involved in neurological processes. The study genotyped 67 Pandemrix-associated narcolepsy patients in Sweden and compared them with what is known about the genetic profile of non-vaccine-related narcolepsy patients (Bomfim et al., 2014). These observations positioned vaccine-associated narcolepsy as *overlapping* and *entangled* with non-vaccine-related narcolepsy.

Another central question was whether the H1N1 virus played a role in the onset of narcolepsy. Both influenza A viruses and streptococcal bacterial infections had been previously linked to neurological conditions such as narcolepsy and Guillain-Barré syndrome. One study (Han et al., 2011) suggested that this was the case in Beijing, where the incidence of narcolepsy had increased in the absence of pandemic vaccines. Some studies from Europe contradicted this conclusion. For example, Melén et al. (2013) traced antibodies against the 2009 H1N1 strain among narcolepsy patients vaccinated with Pandemrix. Focusing on antibodies to a protein present in the H1N1 virus but left out from the vaccine, the authors showed that most of the vaccine-associated narcolepsy patients lacked these antibodies and had thus not contracted the pandemic virus.

As to why Pandemrix triggered narcolepsy, initial reports pointed tentatively to the adjuvant, AS03, which was not included in the other pandemic vaccines used in Europe, such as Celvapan and Focetria. Interestingly, this initial framing resonated with the long-standing suspicion of toxic substances, such as thimerosal or formaldehyde, in cultural discourses around vaccines. Adjuvants are agents that boost the immune reaction, thereby enabling a longer lasting immunity and the use of smaller amounts of viral material in a single shot, an important advantage in vaccine production within the pressing pandemic timeline. The AS03 adjuvant was discussed as a possible culprit in initial responses such as Nohynek et al.’s (2012) report of narcolepsy in Finland. The authors called for further study of adjuvants, as “[a]nimal models have suggested that squalene [a component of the AS03 adjuvant], although at higher doses than used in human vaccines, is capable of contributing to the development of autoimmunity” (Nohynek et al., 2012: 8). Not surprisingly, ideas of a possibly harmful external agent in Pandemrix circulated widely in the media and online discussion forums (e.g. Ilta-Sanomat, 2010; Kelland, 2013; YLE—The Finnish National Broadcasting Company, 2011; see also Hall and Wolf, 2019).

Biomedical attention shifted soon to other material processes and agents. The emerging research suggested that vaccine-associated narcolepsy was a complex phenomenon that could not be captured by the binaries of external/internal and natural/unnatural prominent in cultural discourses of vaccines. Crucial evidence came from GSK’s other AS03 adjuvanted pandemic vaccine, Arepanrix, used in Canada, which had not been linked to narcolepsy. A 2014 paper by Vaarala et al. suggested that, despite having the same components as Arepanrix, Pandemrix had considerably higher amounts of structurally altered viral nucleoprotein. Furthermore, children with Pandemrix-associated narcolepsy had higher levels of antibodies to the nucleoprotein than vaccinated children without narcolepsy, and the presence of antibodies correlated with the presence of the DQB1*06:02 risk allele (Vaarala et al., 2014). The results were supported by a human–mouse comparison: high levels of antibodies to the viral nucleoprotein in DQB1*06:02 transgenic mice vaccinated with Pandemrix. Another vaccine comparison, this time between Pandemrix and Focetria—a pandemic vaccine by Novartis that included a different adjuvant and lower levels of nucleoprotein—provided further evidence for the crucial role of the nucleoprotein (Ahmed et al., 2015).

This line of research pointed to *molecular mimicry*. The hypothesis maintained that the viral nucleoprotein in Pandemrix may create an autoimmune reaction against hypocretin-receptors, which smaller amounts of nucleoprotein could not achieve. Analyzing sera from narcolepsy patients and control groups, Ahmed et al. (2015) proposed “a mechanism for influenza infection/pandemic vaccine-associated narcolepsy”:

In subjects with genetic susceptibility to narcolepsy, the presentation of NP [nucleoprotein] antigen during infection or after immunization with adjuvanted influenza vaccines containing increased amounts of NP may generate high titers of NP antibodies that can persist in the systemic circulation for months. During this time period, either the high titers of NP antibodies or inflammation related to an unrelated infection [for example, streptococcus] may alter the blood-brain barrier, allowing NP antibodies to cross-react with neural tissue expressing HCRT [hypocretin] receptors. (p. 7)

Intriguingly, both the adjuvant and infection still play a role as possible contributing factors. This posits the mechanisms of causation as plural, as several different chains of events involving adjuvants, nucleoproteins, and H1N1 infection may materialize as narcolepsy. This multiplicity was invoked in further studies that identified additional contributing mechanisms to the onset of narcolepsy (e.g. Mosakhani et al., 2017; Saariaho et al., 2015).

These examples show that scientists needed to design complex experiments to track an *invisible* mechanism of injury. As Feltelius et al. (2015) emphasize, “The small hypothalamic area in the brain is not accessible for study *in vivo* during the active phase. Patients may display symptoms and signs long after the potential immune activation” (p. 341). Like epidemiological studies, biomedical research designs engaged in tinkering. They brought together a range of entities, including different vaccines, adjuvants, genetically altered mice, protein structures, control groups, hypocretin levels, and a genetic risk allele. These explorations complicated cultural ideas of vaccines as straightforwardly toxic substances. At the same time, processes inside the body became increasingly seen as active participants in disease causation rather than passive objects of an external threat (a toxic vaccine). As vaccine-associated narcolepsy emerged as a materially complex phenomenon, questions of risk and responsibility were also rendered increasingly multifaceted.

## Negotiating tensions and ambiguities of injury

These tensions and multiplicities in epidemiological and biomedical knowledge were negotiated and mobilized when families formed patient organizations and networks. I focus here on the public websites of three national patient organizations: Narkolepsiföreningen in Sweden; Narcolepsy UK in the United Kingdom; and SOUND—Sufferers of Unique Narcolepsy Disorder in Ireland. Looking at these organizations is illuminating because they employ both shared and distinct strategies while negotiating different dynamics of state recognition of injury. I also refer to the Finnish association TATU for children and adolescents with disabilities and illness resulting from accident or medical injury, which managed state-funded peer support in Finland. However, as the TATU website does not engage openly in activism, I focus on the Swedish, Irish, and UK organizations, which demonstrate how ambiguities of vaccine-associated narcolepsy emerge as a site of politics.

The patient organizations position themselves differently in relation to non-vaccine-related narcolepsy. Narcolepsy UK is a general narcolepsy organization founded in the 1980s with well-established structures. The organization took up vaccine-associated narcolepsy actively: their news section includes repeated posts about legal cases and parliamentary debates involving families affected by vaccine-associated narcolepsy in the United Kingdom. Because of the general narcolepsy framework, vaccine-associated narcolepsy appears closely aligned with non-vaccine-related narcolepsy. In Sweden and Ireland, organizations were formed around vaccine-associated narcolepsy as a specific condition: both Narkolepsiföreningen and SOUND were established by parents of children who developed narcolepsy after Pandemrix vaccination. Narkolepsiföreningen is clearly the bigger of these organizations with 968 members in 2018, while SOUND states that it is “run by a very small team of volunteers who’s family have been impacted” (Narkolepsiföreningen, 2018; SOUND, n.d.b). In the case of the Finnish TATU, Pandemrix-associated narcolepsy was distanced furthest from non-vaccine-related narcolepsy, as TATU supports families affected by accidents and medical injury. The three organizations can all be situated under the phenomena of biosociality and biosocial citizenship theorized by Rabinow (1996), Rose and Novas (2004), Gibbon and Novas (2008), and Navon and Eyal (2014) as characteristic of contemporary biomedicine. However, the differences between the organizations suggest that preexisting structures shape what kinds of communality and activism may arise in different national, cultural, and public health contexts. At the same time, different ways of framing vaccine-associated narcolepsy do not automatically translate into political positions. This is demonstrated by the Finnish organization’s website. Although TATU connects narcolepsy to medical injury, the focus is on peer support, and the organization has worked closely with state actors, managing a publicly funded support project, ALUVE, for families affected by vaccine-associated narcolepsy, and maintaining the service portal Palvelupolkumalli (TATU, 2019).

Different organizational structures and strategies point to inherent challenges in advocating for a previously unknown condition. Should the new condition be aligned with an already recognized condition? Should patients work with state actors, or challenge them, to receive recognition and adequate care? Social scientists working on rare diseases have argued that establishing new conditions in need of care requires complex acts of converging and distancing. These may include establishing sameness with other conditions while also claiming uniqueness; they may also include questioning the very terms of “rareness” (e.g. Rabeharisoa et al., 2014a, 2014b). These concerns are reflected in narcolepsy patient organizations. For example, the name of the Irish organization—Sufferers of Unique Narcolepsy Disorder—highlights the unprecedented nature of vaccine-associated narcolepsy, a strategy that invokes a sense of urgency. Interestingly, however, none of the analyzed organizations draws extensively on general vaccine criticism. The only openly criticized vaccine is Pandemrix, which, the organizations maintain, was licensed too quickly, underwent only “minimal clinical trials,” and lacked long-term follow-up studies (Narcolepsy UK, n.d.a).

A question often debated around vaccine injuries is whether an injury is a temporary adverse reaction, a chronic illness in need of treatment, or a permanent disability (Kirkland, 2016; Reich, 2016). Definitions have legal and financial consequences by justifying or discrediting the need for compensation or therapy. In the case of Pandemrix, patient organizations highlighted that, rather than a sleep disorder, narcolepsy was a *neurological condition affecting the brain*, and thus it impacted all aspects of life: sleep was a mentally exhausting, nearly always-present state in which hallucinations and sleep paralysis took place (e.g. Narcolepsy UK, 2016; see SOUND, n.d.a for a description that highlights the severity of symptoms). Likewise, the organizations’ portrayals of narcolepsy questioned the centrality of measurable symptoms in medical models, as such quantified frameworks omit symptoms such as personality changes, depression, or constant worries around cataplexy attacks (e.g. Narcolepsy UK, 2016). For example, Narkolepsiföreningen’s website highlights variation between people, and emphasizes how extremely exhaustive and scary the symptoms may be (Narkolepsiföreningen, n.d.a).

Specific forms of critique emerged within each organization. In Ireland, critique was directed at the state allowing the use of Pandemrix as a seasonal vaccine still in 2011 when concerns over vaccine-associated narcolepsy were known among public health actors across Europe. In Sweden and the United Kingdom, where compensation schemes had been established, the debate was focused on how to measure severity to determine compensation. Narkolepsiföreningen criticized the state plans of seeing narcolepsy as a 5 percent–20 percent disability equal to the loss of a half or full thumb (Narkolepsiföreningen, 2017). The organization emphasized that Sweden should follow the Finnish and Norwegian guidelines of seeing narcolepsy as similar to brain injuries, thereby highlighting that narcolepsy affected everything the patient did (Narkolepsiföreningen, 2017). Narcolepsy UK actively monitored and protested the state decision to base the level of disability on the moment when the claim was made rather than the likely effects of the injury in the future. The 2015 Upper Tribunal decision, upheld in the Court of Appeal in 2017, to strike down the state policy was welcomed as “recognis[ing] the seriousness of narcolepsy and the magnitude of its effect on those afflicted by it” (Narcolepsy UK, 2015). While engaging in struggles over who controlled discourses of harm and responsibility, these debates were focused on what Pandemrix-associated narcolepsy was as a lifelong injury. As could be expected, patient organizations foregrounded the psychological and interpersonal aspects that had stayed in the margins in the epidemiological and immunological studies other than as numerical scales of sleep patterns, cataplexy attacks, or wakeful hours.

Biomedical aspects of vaccine-associated narcolepsy became a site where questions of accountability were claimed and rejected. Patient organizations maintained that the state had a responsibility to protect its citizens, especially if it assumed compliance with state-recommended vaccinations. Narkolepsiföreningen states poignantly in one of the rotating images on top of the home page: “We vaccinated ourselves against the swine flu. We behaved responsibly” (Narkolepsiföreningen, n.d.b). Britta Lundgren notes that many of those affected by narcolepsy in Sweden contrasted their own responsible behavior with the perceived avoidance of responsibility by state actors (Lundgren, 2017: 33). In particular, patient organizations challenged the invocation of the risk allele DQB1*06:02, as it sidetracked the discussion from the responsibility of the state or GSK to patients themselves. In the biomedical literature, Feltelius et al. (2015), for example, raised the possibility that “vaccine exposure may accelerate the course of subclinical disease” (p. 350), that is, narcolepsy may have been a preexisting disease that had been simply asymptomatic before vaccination. Like many other biomedical articles, the article refers to the vaccine as an “environmental trigger” rather than a causative agent (Feltelius et al., 2015: 341). Lundgren accounts how “some parents or young people have met the argument that ‘you would have gotten narcolepsy later on anyway’” (Lundgren, 2017: 35–36). Narcolepsy UK countered such claims by stating that “[i]t is generally not the case that narcolepsy runs in families” and portrayed the allele as “a case of lower risk for those people who do not have that gene, rather than higher risk for those who do” (Narcolepsy UK, n.d.b). This demonstrates that genetic knowledge is deployed in multiple ways in struggles for recognition and justice. Scholars have shown that parents often welcome genetic knowledge to establish the material realness of a rare disease or a phenotypically blurry condition, or to remove blame from the family (Navon, 2011; Navon and Eyal, 2014). Vaccine-associated narcolepsy differed in this respect, as genetic factors were perceived as potentially moving responsibility away from the state and the pharmaceutical industry.

These examples show how the patient organizations’ concerns took shape in tandem with the growing biomedical literature. In addition to the role of genetics, another question centered on whether Pandemrix had triggered narcolepsy cases that were still asymptomatic—that is, cases of *future narcolepsy*. This was a concern both in the media and in the biomedical literature during the immediate post-pandemic period. One biomedical study noted retrospectively in 2017 that it appeared that “the risk window for the vaccine-induced narcolepsy was as long as two years” (Sarkanen et al., 2018: 183). Interestingly, this type of biomedical uncertainty around embodied processes provides an affective resource for activism by extending the societal significance of the condition. Uncertainty demanded a public recognition that the currently known number of cases was unlikely to reflect the number of cases in the years to come. This dynamic is visible, for example, in a recent article in *The Irish Independent*, in which the journalist as well as an interviewed representative of SOUND invoke the possibility of further undocumented cases—9 years after the pandemic and 7 years after Pandemrix was used as a temporary replacement for the 2011 seasonal influenza vaccine in Ireland (Riegel, 2018).

Finally, the patient organizations’ websites highlighted the high toll of vaccine-associated narcolepsy among children. The Irish, Swedish, and UK websites all include or link texts or videos describing the lives of children and teenagers: these examples typically build on a binary between a painful, monotonous, sometimes unbearable present, and the future that the child was expected to have some day as a healthy adult (e.g. Narcolepsy UK, 2016; SOUND, 2017). On the websites and in linked materials, vaccine-associated narcolepsy emerges as structured on currently likely and previously possible futures: *life-before-Pandemrix, life-after-Pandemrix,* and, most poignantly, *life-that-would-have-been-without-Pandemrix*. While not generally anti-vaccine, these narratives of children and futures echo the rhetoric familiar from vaccine criticism. In such rhetoric, the futures at stake appear embedded in the moment of vaccination, when the injected substance interrupts not only the bodily functions of the vaccinated child, but also the very possibility of a carefree, exciting, effortlessly unfolding future (e.g. Gottlieb, 2016; Reich, 2016). However, these portrayals of children and futures are also shaped more generally by the history of vaccine safety debates, in which children have epitomized questions of state and parental responsibility as the ones who do not make vaccination decisions but need protection (Conis, 2015). In the case of Pandemrix, this dynamic became invested with a heightened poignancy when it became clear that the 2009 pandemic was milder than initially expected. As a result, the image of the pandemic as a threat to children and their futures was replaced by the image of Pandemrix as a threat to children and futures.

## Conclusion

The article has traced the emergence of a novel vaccine injury at the intersection of biomedical, material, social and political worlds. It has approached vaccine injury as a process that takes place through epidemiological and biomedical studies and the public engagements of patient organizations. The case is particularly interesting for theorizing vaccine injuries because of its uniqueness: few recent vaccine injury claims have been established on the population level. The case provides a viewpoint into what happens to a novel vaccine injury after its public recognition. The analysis has shown how vaccine-associated narcolepsy, even when acknowledged by public health and state actors, remains an unfixed and contested object.

I have argued that the ambiguities around vaccine-associated narcolepsy become a site of political and cultural negotiations over responsibility. In particular, risk, harm, and justice are negotiated in relation to the conceptual frameworks of past vaccine injury debates, and non-vaccine-related narcolepsy. These phenomena are echoed and mobilized in multiple and sometimes contradictory ways in the epidemiological and biomedical studies and on patient organization websites. These debates center on the nature of vaccine-associated narcolepsy as a biomedical, lived, and politically significant entity.

The debates about vaccine-associated narcolepsy have considerable implications for public trust in vaccine safety as well as future pandemic preparedness plans (Lundgren and Holmberg, 2017). I conclude by highlighting one key implication: the personalization of risk. As the article has shown, biomedical research on vaccine-associated narcolepsy drew attention to the myriad invisible molecular, neurological, and immunological differences between humans that position us differently in terms of risk for vaccine-associated narcolepsy and, presumably, other autoimmune diseases. This raises several questions: Whose risk matters in the evaluation of vaccine safety? What is acceptable risk, if risk is dispersed differently across the population? Interestingly, this reconfiguration of risk resonates with the idea of each child as immunologically unique often emphasized in vaccine critical discourse (Reich, 2016). In the case of Pandemrix, the idea of situated risk materialized in detailed descriptions of embodied factors and processes. These unintended resonances between scientific evidence of individualized risk of vaccine-associated narcolepsy and the assumption of individualized risk in vaccine critical discourse are an issue that immunization campaigns will need to tackle in order to negotiate questions of trust in public health. This is especially the case with future pandemic vaccines, which are likely to be developed without time for extensive, long-term population-level studies.
